# A Cross-Sectional and Prospective Comparison of Medicinal Cannabis Users and Controls on Self-Reported Health

**DOI:** 10.1089/can.2019.0096

**Published:** 2021-12-09

**Authors:** Nicolas J. Schlienz, Ryan Scalsky, Erin L. Martin, Heather Jackson, Joel Munson, Justin C. Strickland, Marcel O. Bonn-Miller, Mallory Loflin, Ryan Vandrey

**Affiliations:** ^1^ Department of Community Health and Health Behavior, University at Buffalo, Buffalo, New York, USA.; ^2^ University of Maryland School of Medicine, Baltimore, Maryland, USA.; ^3^ Department of Neuroscience, Medical University of South Carolina, Charleston, South Carolina, USA.; ^4^ Realm of Caring Foundation, Colorado Springs, Colorado, USA.; ^5^ Department of Psychiatry and Behavioral Sciences, Johns Hopkins University School of Medicine, Baltimore, Maryland, USA.; ^6^ Department of Psychiatry, University of Pennsylvania Perelman School of Medicine, Philadelphia, Pennsylvania, USA.; ^7^ Center of Excellence for Stress and Mental Health, VA San Diego Health care System, La Jolla, California, USA.; ^8^ Department of Psychiatry, University of California San Diego, School of Medicine, La Jolla, California, USA.

**Keywords:** cannabinoid therapy, cannabis, health, medicinal cannabis, quality of life

## Abstract

**Introduction:** Despite widespread legalization, the impact of medicinal cannabis use on patient-level health and quality of life (QOL) has not been carefully evaluated. The objective of this study was to characterize self-reported demographics, health characteristics, QOL, and health care utilization of Cannabis Users compared with Controls.

**Methods:** A longitudinal, cross-sectional web-based survey study was completed between April 2016 and February 2018. Study participants (*n*=1276) were a convenience sample of either patients with a diagnosed health condition or caregivers of a patient with a diagnosed health condition registered with the Realm of Caring Foundation (a nonprofit organization dedicated to therapeutic cannabis research and education). Participants were invited through e-mail to complete follow-up assessments every 3 months with 33% of participants completing one or more prospective follow-ups. Assessments included self-reported demographics, health care utilization, medication use, pain, anxiety, depression, sleep, and QOL. Cannabis Users (*n*=808) were compared with Controls (*n*=468) using negative binomial regression and linear mixed effects models testing the effect of initiation, cessation, and maintenance of medicinal cannabis use.

**Results:** Cannabis Users self-reported significantly better QOL [*t*(1054)=−4.19, *p*<0.001], greater health satisfaction [*t*(1045)=−4.14, *p*<0.001], improved sleep [children: *t*(224)=2.90, *p*<0.01; adults: [*t*(758)=3.03, *p*<0.01], lower average pain severity [*t*(1150)=2.34, *p*<0.05], lower anxiety [*t*(1151)=4.38, *p*<0.001], and lower depression [*t*(1210)=5.77, *p*<0.001] compared with Controls. Cannabis Users reported using fewer prescription medications (rate ratio [RR]=0.86; 95% confidence interval [CI]: 0.77–0.96) and were less likely to have a past-month emergency department visit (RR=0.61; 95% CI: 0.44–0.84) or hospital admission (RR=0.54; 95% CI: 0.34–0.87). Controls who initiated cannabis use after baseline showed significant health improvements at follow-up, and the magnitude of improvement mirrored the between-group differences observed at baseline.

**Conclusions:** Cannabis use was associated with improved health and QOL. Longitudinal testing suggests that group differences may be due to the medicinal use of cannabis. Although bias related to preexisting beliefs regarding the health benefits of cannabis in this sample should be considered, these findings indicate that clinical trials evaluating the efficacy of defined cannabinoid products for specific health conditions are warranted.

## Introduction

The legalization of cannabis for medicinal use without clinical trials to demonstrate safety and efficacy is unprecedented, yet widespread, and presents significant regulatory challenges.^1^ In the United States, more than 2.1 million individuals are registered with state medicinal cannabis use programs and use cannabis for over 40 different health conditions.^2,3^ The cannabis plant consists of hundreds of distinct chemicals, 120 of which are unique to the cannabis plant (i.e., phytocannabinoids).^4,5^

The two most prevalent phytocannabinoids are Δ^**9**^-tetrahydrocannabinol (THC) and cannabidiol (CBD). THC produces many of the hallmark effects associated with cannabis intoxication (e.g., euphoria, increased appetite, dry mouth, paranoia, cognitive impairment), and is believed to drive the abuse liability of cannabis.^6^ CBD, in contrast, does not produce THC-like intoxicating effects, has low/no abuse liability, and has been associated with relatively few acute adverse effects in human clinical trials, although additional safety data in longer duration trials and populations without a large number of concomitant medications are desired.^7,8^

Although specific pharmaceutical formulations of both THC (for anorexia associated with weight loss in patients with AIDS, or nausea and vomiting associated with cancer chemotherapy) and CBD (Dravet syndrome or Lennox/Gastaut syndrome) have been approved by the Food and Drug Administration (FDA), demand for alternative cannabis products has proliferated. Cannabis legalization has yielded a retail market of products that vary by formulation (e.g., dried flowers, cannabis oils/tinctures intended for oral ingestion, cannabis-infused food and beverage products, concentrated extracts, and topical/transdermal products), method of administration (e.g., smoked, vaporized, swallowed), and chemical composition (e.g., THC-dominant, CBD-dominant, or balanced THC/CBD).

The public health ramifications of medicinal cannabis legalization warrant considerable attention and investment. Prior studies have documented modest increases in cannabis use at the population level following legalization, particularly among older adults, despite a decrease in the rate of Cannabis Use Disorder among users.^9,10^ Mixed results have been observed with respect to opioid-sparing effects of medicinal cannabis legalization.^11–14^ Epidemiological studies indicate that motor vehicle accidents and emergency department (ED) visits have increased within states that have legalized cannabis, although the relatedness of cannabis legalization to these changes continues to be debated.^15,16^ Importantly though, the impact of medicinal cannabis legalization at the level of individual cannabis users is poorly understood.

Observational research methods are ideally suited to evaluate the health effects of medicinal cannabis use broadly, and provide a pathway for identifying specific health conditions and/or cannabis product characteristics that warrant additional study through traditional drug development approaches (i.e., randomized, controlled trials).^17,18^ Prior studies have examined the health and demographic characteristics of medicinal cannabis users, and/or described cannabis product selection or use behaviors among medicinal cannabis users,^19–23^ but have lacked an adequate control group. The aim of the present study was to compare a large convenience sample of medicinal cannabis users to a control group of individuals considering medicinal cannabis use on self-reported measures of health. Additional prospective, longitudinal comparisons within a subsample of cannabis users and controls evaluated the impact of initiation, cessation, and maintenance of medicinal cannabis use on standardized health measures.

## Methods

### Research setting

This study was conducted by the Realm of Caring Foundation (Colorado Springs, CO), a nonprofit organization dedicated to therapeutic cannabis research and education, in collaboration with the Johns Hopkins University School of Medicine (Baltimore, MD). The Realm of Caring Foundation is a resource for those seeking information related to the use of cannabis for therapeutic purposes. Participants were recruited from the Realm of Caring Foundation patient registry and social media posts made by the organization. These participants included both those who were already using a cannabis product and those considering initiation of medicinal cannabis product use. Participants completed assessments through web-based surveys (Qualtrics, Provo, UT). The study was approved by the Johns Hopkins University IRB and informed consent was obtained as part of the survey.

### Participants

Participants (*n*=1276) were enrolled between April 2016 and February 2018. Of these, 524 were adult patients who used cannabis for medicinal purposes and 284 were adult caregivers of children or dependent adults who used cannabis for medicinal purposes (Cannabis Users; *n*=808). The control group consisted of 271 adult patients who were considering, but had not yet initiated therapeutic use of cannabis, and 197 adult caregivers who were considering therapeutic use of cannabis for a dependent child or adult patient (Controls; *n*=468). All participants self-reported that they or their dependent patient had a diagnosed health condition at the time of the baseline assessment. Note that we differentially refer to participants (those who completed the study assessments) and patients (individuals with health conditions) throughout the article.

### Study measures

Participants completed a web-based survey that measured several content areas. Adult patients who were capable self-reported all information. Caregivers completed study assessments based on observations of the dependent patient, either adult or child, under their care. Demographic information included the age, sex, race/ethnicity, place of residence, marital status, and highest level of education completed (limited to patients age 18 and over) of the patient. The primary diagnosed health condition for which the patient was using, or considering medicinal cannabis use, was recorded. Daily dose, frequency of use, and route of administration were recorded, to the extent possible, for current prescription medication, over-the-counter (OTC) medication, and cannabis products. Participants reported past-month outpatient health care visits, ED visits, and hospital admissions, as well as past-month sick days taken from work/school. Validated assessments were used to assess past-month quality of life (World Health Organization Quality-of-Life assessment; score-range: 1–5 for individual items and 4–20 for composite scores; WHOQOL-BREF^24^), pain (Numeric Pain Rating Scale; score-range: 0–10; NPRS^25^), anxiety and depression (Hospital Anxiety and Depression Scale; score-range: 0–21; HADS^26^), and sleep (Pittsburgh Sleep Quality Index^27^ [PSQI]) for adults; score-range: 0–21; Children's Sleep Habits Questionnaire-Abbreviated^28,29^ (CSHQ-A) for children; score-range: 22–110.^a^ Participants who completed the survey each month were entered into a raffle to win one of twenty $50 gift cards.

Following baseline survey completion, participants were prompted through e-mail to complete follow-up assessments at 3-month intervals. Approximately one-third of participants completed at least one follow-up assessment (32.6%) at an average of 284 days following baseline. On average, these participants completed 1.8 assessments (median=1).

### Data analyses

Patients were classified as Cannabis Users or Controls based on self- or caregiver-reported current cannabis use at baseline. There were no differences in study findings when controlling for self-reported versus caregiver report, therefore results are presented without categorizing by data source. Descriptive statistics were used to summarize patient demographics, primary health condition, and cannabis use characteristics. Independent-samples *t*-tests and chi-square tests of independence were used to examine differences between Cannabis Users and Controls for continuous and categorical variables, respectively. For count variables (i.e., medication use, outpatient visits, ED visits, hospital admissions, sick days from work/school), negative binomial regressions were conducted. The Benjamini/Hochberg procedure^30^ was used to control Type I error associated with multiple comparisons. All results remained significant after controlling for the false discovery rate. Longitudinal data (all completed follow-up assessments) were analyzed using linear mixed effects model parameterizing a between- and within-person effect of medicinal cannabis use on health symptoms^31^ as well as accounting for the effect of time independent of medicinal cannabis use status (see Supplementary Materials for additional details about this methodology). These models differentiated and tested the (1) relation of the average prevalence of medicinal cannabis use reported throughout the analyzed period (between-subject effects) and (2) the relation of time-specific deviations in medicinal cannabis use on health symptoms (e.g., initiation or cessation of use; within-subject effects). Longitudinal analysis of CSHQ-A scores was not conducted due to the low number and density of follow-up assessments for patients under age 18 (*n*=114 follow-ups from 77 participants) as well as the infrequent report of cannabis cessation among children in the Cannabis User group (*n*=3). Inclusion of patient demographic (age, gender, race) and assessment (self-report vs. observer report) covariates in mixed effects model did not alter the pattern of findings or their significance. All models were conducted in *R* statistical language using the *nlme* package.^32^

## Results

### Patient characteristics

Cannabis Users and Controls were similar on major demographic characteristics (Table 1). Patients were predominantly Caucasian (79%), female (63%), and most patients age 18 or older had a greater than high school education. Cannabis Users were significantly older than Controls [*t*(1274)=−2.31, *p*<0.05], with a mean age of 38 versus 35 years, respectively, and were more likely to report a history of nontherapeutic cannabis use (e.g., past-month nontherapeutic cannabis use was 10% vs. 5%, respectively).

**Table 1.** Participant Demographics

	Cannabis users (*n*=808)	Controls (*n*=468)	*p*
Age			
Mean (SD)	38 (20)	35 (21)	<0.05
Range; *n* (%) below age 18	1–86; 175 (22)	1–82; 136 (29)	
Sex, *n* (%)			
Male	298 (37)	177 (38)	0.71
Female	510 (63)	291 (62)	
Race, *n* (%)			
Caucasian	637 (79)	372 (79)	0.48
African American	16 (2)	18 (4)	
Hispanic/Latino	38 (5)	27 (6)	
Other	75 (9)	43 (9)	
Not reported	42 (5)	11 (2)	
Education (among age ≥18), *n* (%)			
High school or less	106 (17)	68 (20)	0.05
Some college	133 (21)	78 (23)	
Undergraduate degree	183 (29)	87 (26)	
Graduate degree	123 (19)	56 (17)	
Trade/technical training	51 (8)	30 (9)	
Not reported	37 (6)	13 (4)	
Nontherapeutic cannabis use, *n* (%)			
Lifetime	250 (31)	113 (24)	0.01
Past year	111 (14)	42 (9)	0.01
Past month	79 (10)	24 (5)	0.005

SD, standard deviation.

### Primary health condition

The primary self-reported diagnosed health condition for which participants used cannabis, or considered cannabis use, was categorized into one of seven broad health categories (Table 2) due to the incredible diversity of diagnosed health conditions reported by study participants. Cannabis Users and Controls did not significantly differ with respect to type of primary health condition (*χ^2^* [6, *n*=1274]=7.77, *p*=0.35). The most frequently endorsed health conditions were neurological (e.g., epilepsy, multiple sclerosis), chronic pain (e.g., fibromyalgia, chronic back pain), and psychiatric (e.g., anxiety, depression, post-traumatic stress disorder) disorders.

**Table 2.** Primary Medical Condition for Which Participants Were or Were Not Considering Use of Cannabis

	Cannabis users (*n*=808)	Controls (*n*=468)	*p*
Primary medical condition			0.35
Neurological, *n* (%)	307 (38)	170 (36)	—
Chronic pain, *n* (%)	204 (25)	108 (23)	—
Neuropsychiatric, *n* (%)	146 (18)	94 (20)	—
Autoimmune, *n* (%)	75 (9)	46 (10)	—
Cancer, *n* (%)	59 (7)	33 (7)	—
Insomnia, *n* (%)	6 (1)	10 (2)	—
Other, *n* (%)	11 (2)	7 (1)	—

### Cannabis use characteristics: product recommendations, type, and dose

Among Cannabis Users, 27% reported that a physician explicitly recommended cannabis use; 45% said that a physician did not recommend use, and 28% provided no response. Cannabis use was reported to be a first-line therapy for the primary health condition among 11%, a second-line therapy for 18%, an adjunctive therapy for 39%, and a treatment of last resort for 29% of Cannabis Users (3% did not answer).

Fifty-eight percent of patients used CBD-dominant products. By comparison, THC-dominant products were used by 13%, balanced THC/CBD products by 5%, and products in which the highest concentration was a minor cannabinoid, such as cannabigerol (CBG) or cannabinol (CBN), by 3% of Cannabis Users. Many participants (21%) did not know or did not specify the chemotype of the cannabis products they used.

Cannabis tinctures or oils intended for oral ingestion were the most commonly reported cannabis formulations (47%), followed by dried cannabis flowers (9%), “edibles” (8%), concentrates (3%), and other formulations such as topicals or suppositories (3%). Thirty-one percent of respondents did not report the formulation of cannabis product they most often used. Variability in product type, formulation, method of use, and lack of standard dose units, packaging, and labeling made concise summarization of dosing difficult. Certificates of analysis were obtained from manufacturers that enabled daily CBD and THC dose calculations for 353 patients taking specific oral cannabis products. Among those participants, the mean total daily CBD dose was 79 mg (median=40 mg; range=1–1050 mg) and the mean total daily THC dose was 3 mg (median=1.4 mg; range=0.1–40.3 mg). Adjusted for body weight, the mean total daily CBD dose was 1.4 mg/kg (median=0.6 mg/kg; range=0.01–15.7 mg/kg) and the mean total daily THC dose was 0.05 mg/kg (median=0.02 mg/kg; range=<0.01–0.6 mg/kg).

### Baseline health symptoms

Cannabis Users had significantly better symptoms on most self-reported health assessments compared with Controls at baseline (Table 3).

**Table 3.** Group Comparison on Baseline General Health Outcomes

	Cannabis user mean (SD) [*n*]	Controls mean (SD) [*n*]	*p*	Cohen's *d*
WHOQOL-BREF^a^				
Quality-of-life rating	3.5 (1.2) [674]	3.2 (1.2) [382]	<0.001	0.25
Health satisfaction rating	2.4 (1.1) [669]	2.1 (1.0) [378]	<0.001	0.29
Physical health domain	12.1 (3.8) [671]	11.2 (3.6) [376]	<0.001	0.24
Psychological domain	13.3 (3.3) [670]	12.2 (3.3) [377]	<0.001	0.33
Social relationships domain	13.3 (4.0) [656]	12.5 (4.3) [371]	<0.01	0.19
Environment domain	15.0 (3.2) [678]	14.8 (3.0) [384]	0.18	0.06
NPRS^a^				
Average pain	3.9 (2.9) [742]	4.3 (3.0) [410]	<0.01	0.14
Worst pain	5.8 (3.6) [738]	6.1 (3.5) [407]	0.18	0.08
HADS^a^	—	—	—	
Anxiety subscale	9.2 (5.2) [730]	10.5 (5.1) [423]	<0.001	0.25
Depression Subscale	6.7 (4.8) [771]	8.4 (5.1) [441]	<0.001	0.34
CSHQ (child sleep)^a^	—	—	—	
Total score	49.9 (10.6) [129]	54.3 (11.8) [97]	<0.01	0.39
Bedtime resistance	11.4 (4.8) [172]	12.3 (5.1) [142]	0.08	0.18
Sleep onset delay	2.5 (1.0) [177]	3.1 (1.2) [141]	<0.001	0.54
Sleep duration	2.5 (1.3) [178]	2.6 (1.4) [142]	0.73	0.07
Sleep anxiety	6.8 (3.6) [162]	7.1 (3.7) [131]	0.47	0.08
Night wakenings	7.1 (2.5) [163]	8.1 (2.9) [132]	<0.01	0.37
Parasomnias	6.7 (2.2) [153]	7.7 (2.7) [116]	<0.01	0.41
Sleep disordered breathing	2.0 (1.0) [177]	2.1 (1.2) [141]	0.48	0.09
Daytime sleepiness	9.4 (2.5) [178]	9.9 (3.1) [141]	0.08	0.18
PSQI (adult sleep)^a^	—	—	—	
Global score	8.9 (4.2) [501]	9.9 (4.2) [259]	<0.01	0.24
Subjective sleep quality	1.5 (0.9) [618]	1.7 (0.9) [322]	<0.001	0.22
Sleep latency	1.6 (1.1) [630]	1.8 (1.0) [326]	<0.01	0.19
Sleep duration	0.9 (1.1) [630]	1.1 (1.1) [326]	<0.05	0.18
Habitual sleep efficiency	1.0 (1.2) [520]	1.1 (1.3) [273]	0.29	0.08
Sleep disturbances	1.8 (0.6) [628]	1.9 (0.6) [325]	<0.01	0.17
Use of sleep medication	1.1 (1.4) [624]	1.2 (1.4) [317]	0.44	0.07
Daytime dysfunction	1.2 (0.7) [613]	1.1 (0.7) [316]	0.17	0.14

^a^ For the WHOQOL-BREF, higher scores indicate better outcomes, for all other measures, lower scores indicate better outcomes.

CSHQ, Children's Sleep Habits Questionnaire; HADS, Hospital Anxiety and Depression Scale; NPRS, Numeric Pain Rating Scale; PSQI, Pittsburgh Sleep Quality Index; WHOQOL-BREF, World Health Organization Quality of Life-BREF.

#### Quality of life

On the WHOQOL-BREF, Cannabis Users reported greater quality of life (QOL) [*t*(1054)=−4.19, *p*<0.001] and perceived health satisfaction [*t*(1045)=−4.14, *p*<0.001], and had significantly higher composite domain scores for physical health [*t*(1045)=−3.52, *p*<0.001], psychological health [*t*(1045)=−4.87, *p*<0.001], and social relationships [*t*(1025)=−3.05, *p*<0.01], but exhibited comparable environmental health scores compared with Controls [*t*(1060)=−1.36, *p*=0.18].

#### Pain

Compared with Controls, Cannabis Users reported significantly lower average pain in the past month on the NPRS [*t*(1150)=2.34, *p*<0.05], but did not differ on ratings of worst pain in the past month [*t*(11143)=1.33, *p*=0.18].

#### Anxiety and depression

Cannabis Users had significantly lower scores on the Anxiety [*t*(1151)=4.38, *p*<0.001] and Depression [*t*(1210)=5.77, *p*<0.001] subscales of the HADS compared with Controls.

#### Sleep

As assessed by the CSHQ, caregiver reports indicated that Cannabis Users under age 18 had better overall sleep habits [*t*(224)=2.90, *p*<0.01], faster sleep onset [*t*(316)=4.91, *p*<0.001], less frequent night awakenings [*t*(293)=2.99, *p*<0.01], and fewer parasomnias [*t*(267)=3.12, *p*<0.01] compared with Controls under age 18. As assessed by the PSQI, adult Cannabis Users had greater sleep quality [*t*(938)=3.96, *p*<0.001], shorter sleep latency [*t*(954)=3.29, *p*<0.01], longer sleep duration [*t*(954)=2.28, *p*<0.05], fewer sleep disturbances [*t*(951)=2.77, *p*<0.01], and a significantly better PSQI Global Sleep Score compared with adult Controls [*t*(758)=3.03, *p*<0.01].

#### Medications, health care utilization, and sick days

**FIG. 1.**
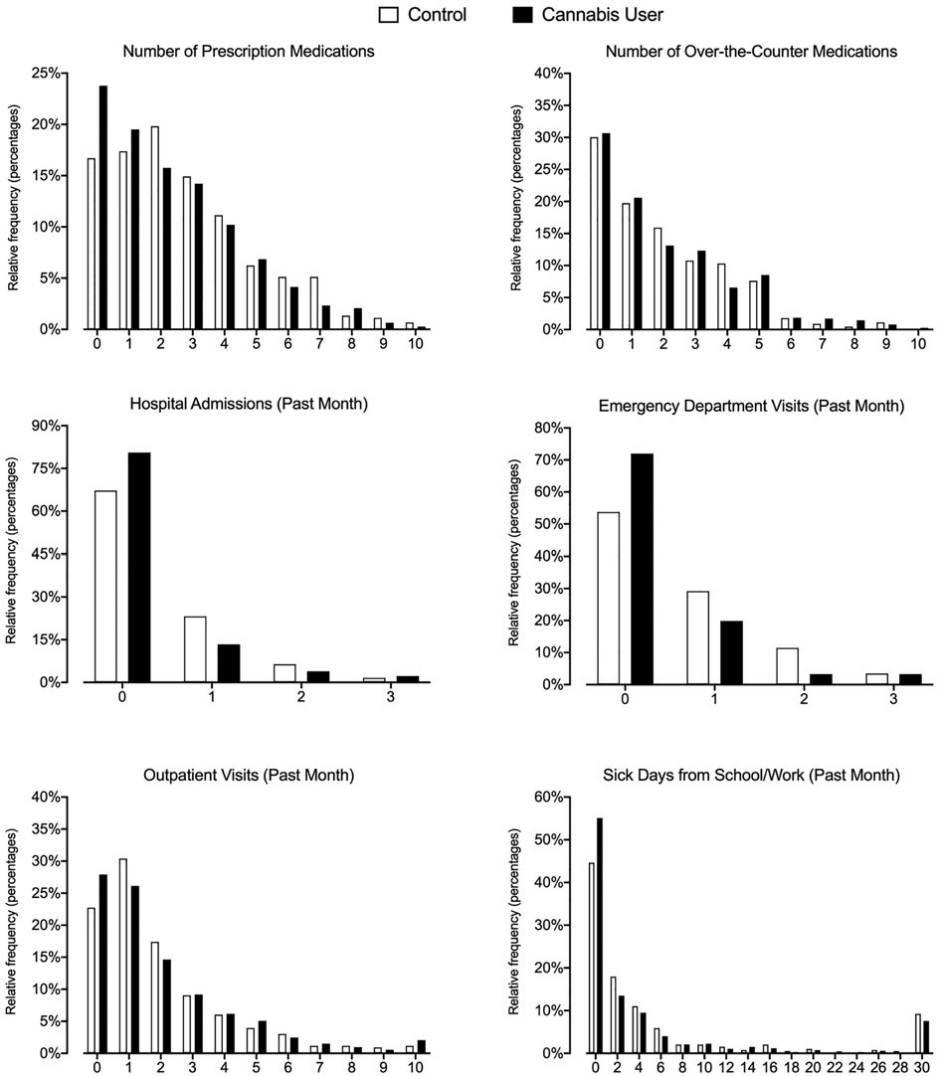
Distribution of medication use and health care utilization by group assignment at baseline.

Results for all negative binomial count data appear in Table 4 and distribution of responses in Figure 1. Cannabis Users reported 14% fewer current prescription medications (95% confidence interval [CI]: 0.77–0.96), 39% fewer past-month ED visits (95% CI: 0.44–0.84), and 46% fewer hospital admissions (95% CI: 0.34–0.87) than the Control group. Groups did not significantly differ in the number of OTC medications, past-month outpatient health care visits, or past-month sick days taken from work/school.

**Table 4.** Group Prediction of Medication Use, Past-Month Health Care Utilization, and Sick Days

Dependent variable, (*n*)	Exp(B)	95% CI	Likelihood ratio χ^2^
Predictor			
Prescription medications, (*n*=774)	—	—	7.71^a^
Group (cannabis users)	0.86^a^	0.77–0.96	—
Over-the-counter medications, (*n*=763)	—	—	0.74
Group (cannabis users)	1.06	0.93–1.22	—
Outpatient visits, (*n*=731)	—	—	0.11
Group (cannabis users)	1.03	0.89–1.19	—
Emergency department visits, (*n*=307)	—	—	9.31^a^
Group (cannabis users)	0.61^a^	0.44–0.84	—
Hospital admissions, (*n*=180)	—	—	6.35^b^
Group (cannabis users)	0.54^b^	0.34–0.87	—
Sick days from school/work, (*n*=675)	—	—	1.86
Group (cannabis users)	0.83	0.63–1.09	—

^a^ *p*<0.01, ^b^*p*<0.05.

CI, confidence interval.

### Longitudinal health symptoms

**FIG. 2.**
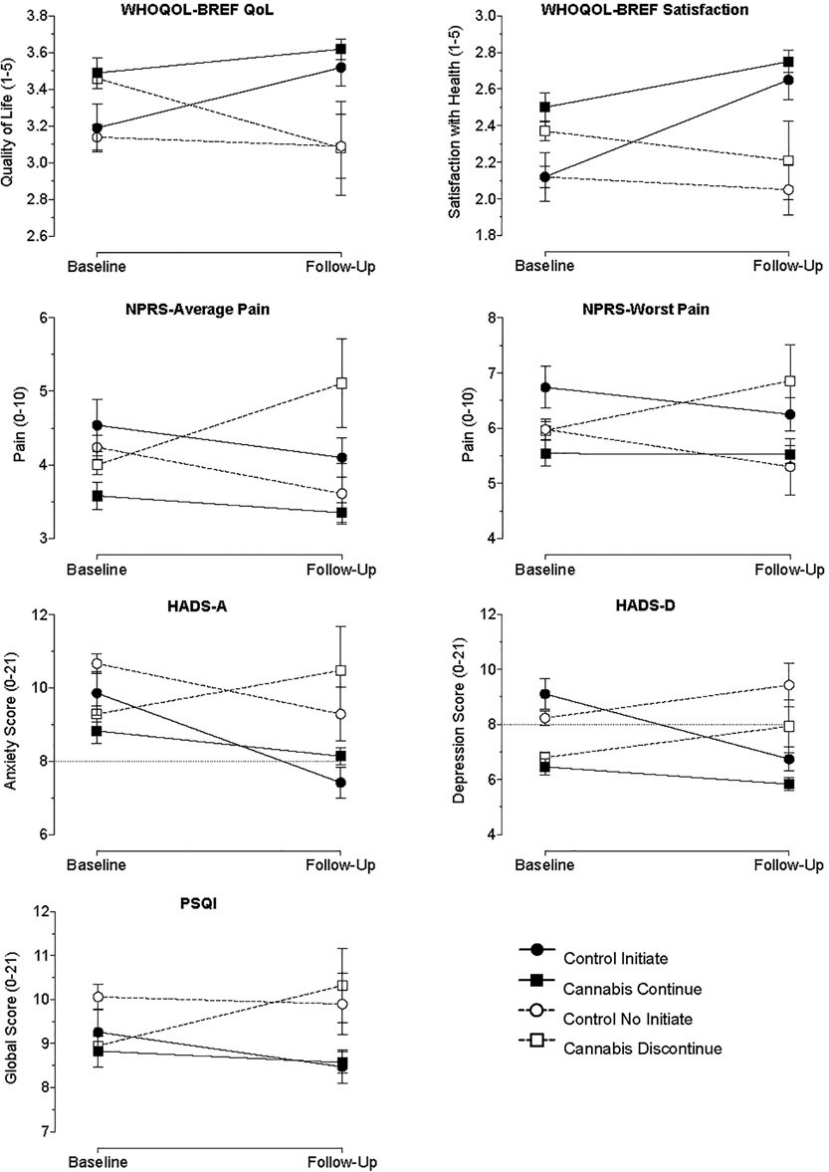
Observed values for health outcomes at baseline and follow-up by medicinal cannabis use status. Plotted are values for the Control group who initiated cannabis use in follow-up (solid circle/solid line), Cannabis User group who continued use in follow-up (solid square/solid line), Control group who did not initiate use in follow-up (open circle/dotted line), and Cannabis Group that discontinued use in follow-up (open square/dotted line). For baseline groupings, individuals who did not provide follow-up data were treated as belonging in the nonuse group for presentation purposes. Y-axis legends reflect total scale range. Dotted line on HADS-A and HADS-D represent clinical cutoff for patient follow-up. Individual sample sizes range from 22 to 523 depending on the time point, group, and assessment depicted. Error bars are standard error. HADS, Hospital Anxiety and Depression Scale.

Raw data for primary health symptoms at baseline and follow-up by medicinal cannabis use status are plotted in Figure 2 (see Supplementary Materials for marginal mean plots and sensitivity analyses evaluating alternative parameterizations). Consistent with the baseline analyses, significant between-person effects of medicinal cannabis use (i.e., average prevalence of medicinal cannabis use reported throughout the analyzed period) were observed for QOL (*b*=0.35, *p*<0.001), perceived health satisfaction (*b*=0.35, *p*<0.001), past-month average pain (*b*=−0.47, *p*<0.05), Anxiety (*b*=−1.58, *p*<0.001), and Depression (*b*=−1.78, *p*<0.001) subscales of the HADS, and PSQI Global Sleep Score (*b*=−1.15, *p*<0.01). These effects reflected better health scores (e.g., lower anxiety) for individuals reporting medicinal cannabis use, on average, across the reported period (i.e., averaging medicinal cannabis exposure over time).

Significant within-person effects of medicinal cannabis use (i.e., association of time-specific deviations in medicinal cannabis use such as initiation or cessation with health symptoms) were also observed for QOL (*b*=0.22, *p*<0.01), perceived health satisfaction (*b*=0.23, *p*<0.01), past-month average pain (*b*=−0.42, *p*<0.05), past-month worst pain (*b*=−0.46, *p*<0.05), and the Anxiety (*b*=−1.56, *p*<0.001) and Depression (*b*=−1.87, *p*<0.001) subscales of the HADS. These within-person effects each reflected improved health scores during specific assessment periods in which medicinal cannabis was reported compared with assessment periods in which it was not reported for individual participants (i.e., indicating that initiation of medicinal cannabis use, on average, was associated with improved symptoms and cessation of medicinal cannabis use, on average, was associated with worse symptoms on these variables).

A sensitivity analysis was conducted to determine if missing data contributed to the observed results. No significant differences between participants providing follow-up data and those without were observed in age (*p*=0.99), gender (*p*=0.54), race (*p*=0.52), report status (self vs. observer) (*p*=0.58), or any of the global health measures analyzed (*p* values >0.16). Participants reporting current medicinal cannabis use at baseline were more likely to provide a follow-up assessment (odds ratio [OR]=1.40, *p*=0.008). Sensitivity models in which only participants with follow-up data were analyzed found between- and within-subject associations in the same direction and significance as the primary models are reported above.

Qualitative information was coded from 67 participants who reported discontinuation at some point during the longitudinal period. Over half (55%) cited financial concerns as a reason for discontinuation. Other reasons for discontinuation included legal/employment restrictions (16%), no benefit (13%), and negative health consequences (13%).

## Discussion

Decisions surrounding medicinal cannabis use are challenging for both clinicians and patients due to the diversity of products, abuse liability of cannabis, potential product contamination or label inaccuracies, complicated regulatory structure, and dearth of controlled clinical trials on defined products for targeted health indications.^1^ In this study, Cannabis Users reported better health and QOL, and less health care utilization compared with largely comparable Controls. The observed clinical benefit associated with medicinal cannabis use in this study is consistent with other observational studies.^19,23,33–35^ That only 27% of participants reported that a physician explicitly recommended medicinal cannabis use is somewhat concerning. Increased physician involvement in medicinal cannabis decision making is desirable for patient safety as well as for monitoring, recording, and disseminating clinical outcomes. This study extends prior research by including a large sample size, both child and adult patients, only individuals who self-reported a diagnosed health problem, assessment of multiple health domains, and a control group.

CBD-dominant products were used at a higher rate relative to THC-dominant products, and doses of CBD (mean 79 mg; 1.4 mg/kg) and THC (mean 3 mg; 0.5 mg/kg) used tended to be lower than what has been used in prior human laboratory studies and clinical trials. For comparison, the recommended maintenance dosage (listed on the package insert) for Epidiolex in the treatment of rare seizure disorders is 10–20 mg/kg/day. The recommended starting dose of dronabinol (listed on the package insert) is 5 mg/day for the treatment of anorexia in adults with HIV/AIDS and is 20–30 mg/day for adults who have nausea or emesis associated with chemotherapy. Of note, most participants in this study were using cannabis for health conditions other than the FDA-approved uses of CBD or THC, and for which effective doses have not been determined in controlled clinical trials.

An additional contribution of this study was the evaluation of prospective changes in health symptoms following initiation, cessation, or maintenance of medicinal cannabis product use. These analyses indicated significant within-person effects, in addition to the between-person effects observed between Cannabis Users and Controls at baseline. Within-person change reflected the observation that initiation of medicinal cannabis use resulted in significant increases in QOL and health satisfaction as well as decreases in anxiety and depression that were comparable in magnitude to the difference between Cannabis Users and Controls observed at baseline. Similarly, maintenance on medicinal cannabis in the Cannabis User group resulted in sustained improvements on these measures, whereas cessation of use often resulted in a decrease in health and QOL indicating that stopping medicinal cannabis use was associated with a diminishing or rebound of effect. Notably, pain and sleep showed less robust impacts when evaluated in this longitudinal setting. This outcome is not entirely surprising given that the cross-sectional comparisons for these measures made at baseline were consistent with smaller magnitude differences. Additionally, evidence for the opioid-sparing effects of cannabis products on moderate or severe chronic pain have been mixed, as noted in the introduction. It is also possible that the magnitude (and potential direction) of these effects will vary depending on the chronic health condition evaluated (e.g., see evidence of specific sleep-related motives for medicinal cannabis use among individuals with PTSD^36^).

Several methodological limitations must be acknowledged. The study was conducted with a convenience sample of individuals registered with the Realm of Caring Foundation who were willing to complete a research assessment; thus, this sample may not be representative of medicinal cannabis users broadly. In addition, biases may be represented in both groups. Specifically, individuals in the cannabis group likely already observed a benefit from use and continued to use medicinal cannabis products for this reason. On the other hand, individuals in the control group were those seeking information regarding medicinal cannabis products, and therefore have both unresolved symptomatology and at least some belief that cannabis may improve those symptoms. High rates of missing data were also observed during the prospective portion of the study. This attrition is likely attributable to study-related factors, including the relatively modest incentives (i.e., probabilistic payments) and the exclusive use of reminder emails to encourage compliance. Sensitivity analyses indicated that individuals with missing data did not significantly differ on demographic factors or the health-related symptoms analyzed and evaluation of longitudinal health symptoms in a completion-only group revealed similar findings. Nevertheless, future efforts to improve retention should be explored (e.g., closer monitoring or an escalating incentive schedule for long-term adherence). Last, the effect sizes observed were generally small in magnitude (most Cohen's *d* 0.20-to-0.30). The clinical significance of these differences are difficult to assess due to reliance on self-report versus clinician assessment or objective measures of health, the heterogeneity of the sample with respect to health condition, the type of cannabis products being used, and the manner in which products were used (dosing regimen as well as physician supervision of use). It is important that additional research on the magnitude and clinical viability of these effects be conducted in more controlled clinical settings in which known and consistent dosing and product types are utilized.

The key finding of this study is that medicinal cannabis use was associated with more positive ratings of health and QOL, assessed across multiple domains. Prospective analyses found that Controls showed improvement in health and QOL if they initiated medicinal cannabis use, and that Cannabis Users showed diminished health and QOL if they stopped cannabis use. Because of this, we hypothesize that these group differences were related to the use of medicinal cannabis products. That said, due to the aforementioned limitations, the results of this study do not provide definitive evidence that cannabis is an effective therapeutic. Rather, the results clearly indicate that additional research should be conducted to further evaluate the observed data in more targeted, representative subpopulations of cannabis users. Such studies can be used to identify specific health conditions, product types, and doses that are appropriate for evaluation in controlled clinical trials. Although the current design included a control group to improve interpretation, future observational studies should consider the use of Ecological Momentary Assessment technology (i.e., repeated sampling of behavior in real time) and/or corroboration of patient self-reports with collateral data from additional sources such as electronic medical records or direct reporting from the physician or other health professional caring for the patient.

## Supplementary Material

Supplemental data

## Abbreviations Used

CBD: cannabidiol
CI: confidence interval
CSHQ-A: Children's Sleep Habits Questionnaire-Abbreviated
ED: emergency department
FDA: Food and Drug Administration
HADS: Hospital Anxiety and Depression Scale
NPRS: Numeric Pain Rating Scale
PSQI: Pittsburgh Sleep Quality Index
QOL: quality of life
RR: rate ratio
SD: standard deviation
THC: tetrahydrocannabinol
WHOQOL-BREF: World Health Organization Quality of Life-BREF

## Notes

^a^
Clinical cutoffs by scale: HADS score of 8 or higher is associated with clinical diagnosis; PSQI score of 5 or higher indicates significant sleep disturbance; there are no widely accepted clinical cutoffs for the WHOQOL-BREF, NPRS, or CSHQ-A.
