# Supplementary material for: A Cross-Sectional and Prospective Comparison of Medicinal Cannabis Users and Controls on Self-Reported Health
Source: Cannabis Cannabinoid Res. 2021 Dec 9;6(6):548–58. doi: 10.1089/can.2019.0096 (PMC8713273; doi:10.1089/can.2019.0096)
Supplement: Supplemental data [file Suppl_Data.pdf]

Supplementary Data

Supplementary Material

Linear mixed effects model parameterization and sensitivity analyses

General linear mixed models were used to evaluate the effects of medicinal cannabis use on the prospectively collected follow-up data. These tests were designed to account for: (1) general effects of medicinal cannabis use, (2) the effect of medicinal cannabis use at specific assessments, and (3) an effect of time that was independent of cannabis treatment. Different parameterizations of these effects were explored and are described below. A consistent conclusion of these parameterizations is a positive effect of medicinal cannabis use on the self-reported health outcomes evaluated in this longitudinal setting similar to those described in the primary article. All models used a random intercept and maximum likelihood estimation. Maximum likelihood estimation was used to help account for missing data at follow-up. No significant differences between participants providing follow-up data and those without were observed in age ($p = 0.99$), gender ($p = 0.54$), race ($p = 0.52$), report status (self vs. observer) ($p = 0.58$), or any of the global health measures analyzed (p values > 0.16). Participants reporting current medicinal cannabis use at baseline were more likely to provide a follow-up assessment (odds ratio [OR] = 1.40, $p = 0.008$). Additional sensitivity analyses described below evaluated only individuals providing follow-up data and revealed a similar pattern of effects in direction and significance.

Person mean centering approach

$$Health\ Outcome_{ij} = \pi_{0i} + (Current\ Cannabis\ Use - Mean\ Cannabis\ Use) * \pi_{1i} + Time(Days) * \pi_{2i}$$

$$\pi_{0i} = \gamma_{00} + Mean\ Cannabis\ Use * \gamma_{01}$$

$$\pi_{1i} = \gamma_{10}$$

$$\pi_{2i} = \gamma_{20}$$

The primary parameterization evaluated the effects of medicinal cannabis use after partitioning these effects into between-subject and within-subject level influences. This partitioning reflected that the association of medicinal cannabis use with health outcomes may be influenced by (1) a relation of the average prevalence of medicinal cannabis use reported throughout the analyzed period (between-subject) and/or (2) a relation of the time-specific deviations in medicinal cannabis use (within-subject). This within-subject effect is critical as it captures the impact of initiation or cessation of cannabis use on health indicators (i.e., assessment-specific fluctuations in cannabis treatment). These nonmutually exclusive influences were included in models by using a person-mean centering approach that divided medicinal cannabis use into a between-subject person mean and within-subject person mean deviation (Wang and Maxwell[S1]). As reported in the primary article, the between-person effects were consistent with the baseline comparisons reflecting overall improvements in the same global health indicators. Within-person effects were also generally consistent with the exception that a modest effect was observed on recent worst pain and that the effect on sleep outcomes was no longer significant at the within-person level. The marginal means for the analyzed model are plotted in Supplementary Figure S1. These outcomes all corresponded to the pattern of effects observed in the raw data. Sensitivity analyses subsetting to only participants with full outcome data showed a similar direction and significance of effect for each of these reported outcomes.

Time-varying cannabis use without average effects

$$Health\ Outcome_{ij} = \pi_{0i} + Current\ Cannabis\ Use * \pi_{1i} + Time(Days) * \pi_{2i}$$

$$\pi_{0i} = \gamma_{00} + BaselineUse * \gamma_{01} + Use\ in\ FU * \gamma_{02} + Use\ in\ FU * BaselineUse * \gamma_{03}$$

$$\pi_{1i} = \gamma_{10}$$

$$\pi_{2i} = \gamma_{20} + BaselineUse * \gamma_{21}$$

An alternative approach assessed time-varying effects of (1) medicinal cannabis use (dichotomous) and (2) time measured in days since baseline. Additional time invariant (level 2) variables included self-reported

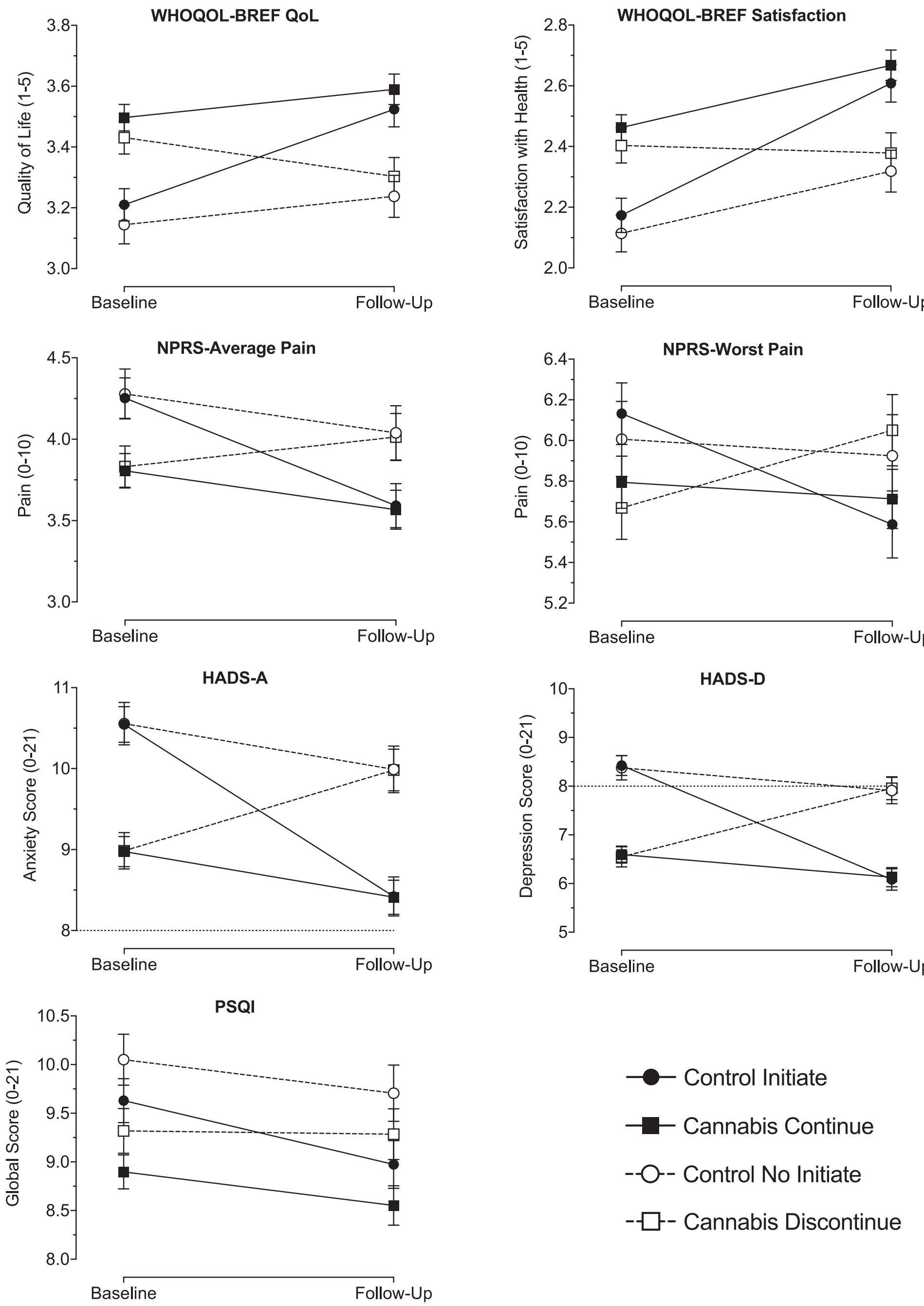

SUPPLEMENTARY FIG. S1. Marginal means for health outcomes at baseline and follow-up by medicinal cannabis use behavior. Plotted are marginal means from the mixed effects model parameterizing person-mean centered effects. Follow-up data are presented at the average time since baseline (284 days). Error bars are standard error.

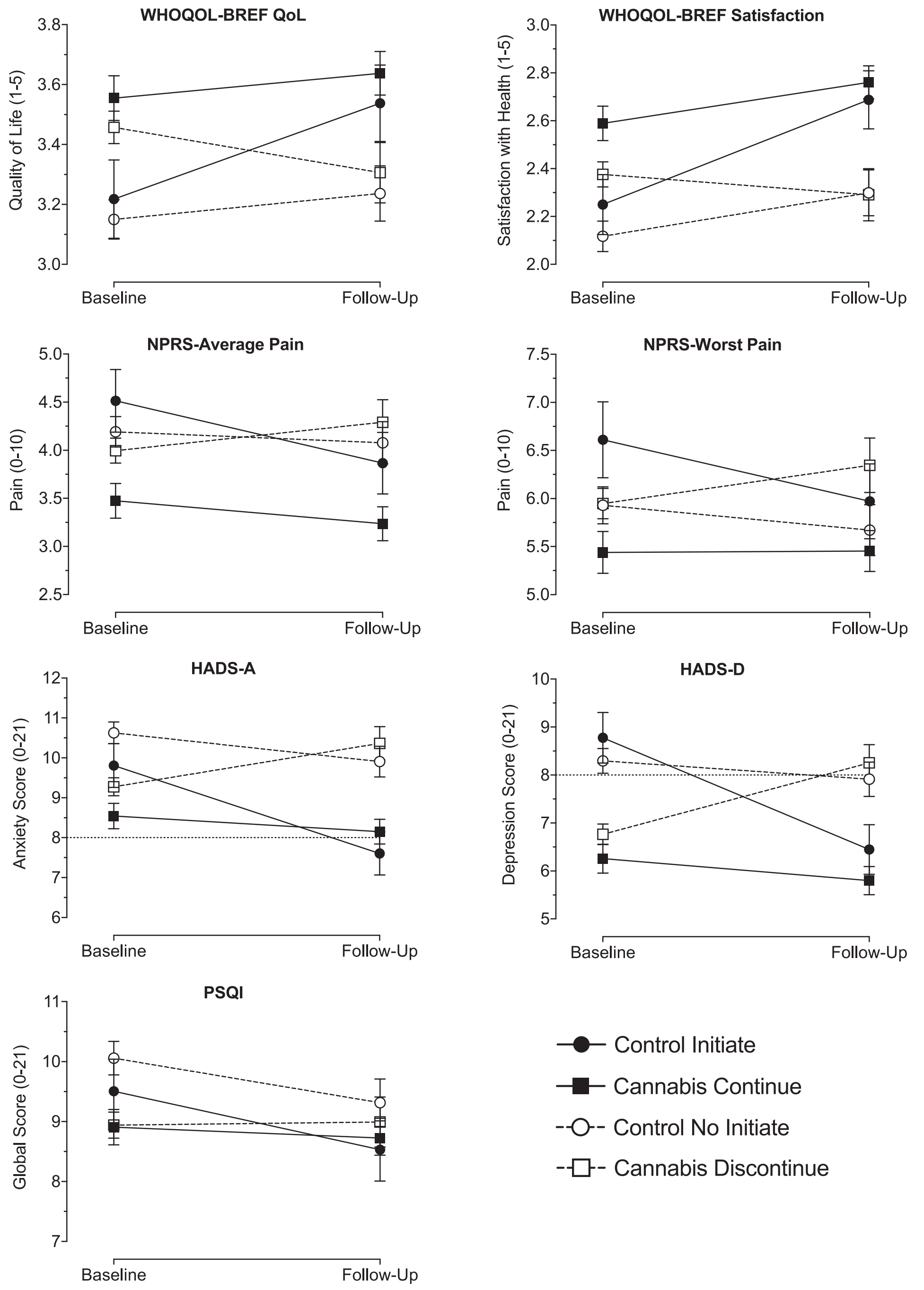

SUPPLEMENTARY FIG. S2. Marginal means for health outcomes at baseline and follow-up by medicinal cannabis use behavior. Plotted are marginal means from the mixed effects model parameterizing cannabis use as time-varying current cannabis use. Follow-up data are presented at the average time since baseline (284 days). Error bars are standard error.

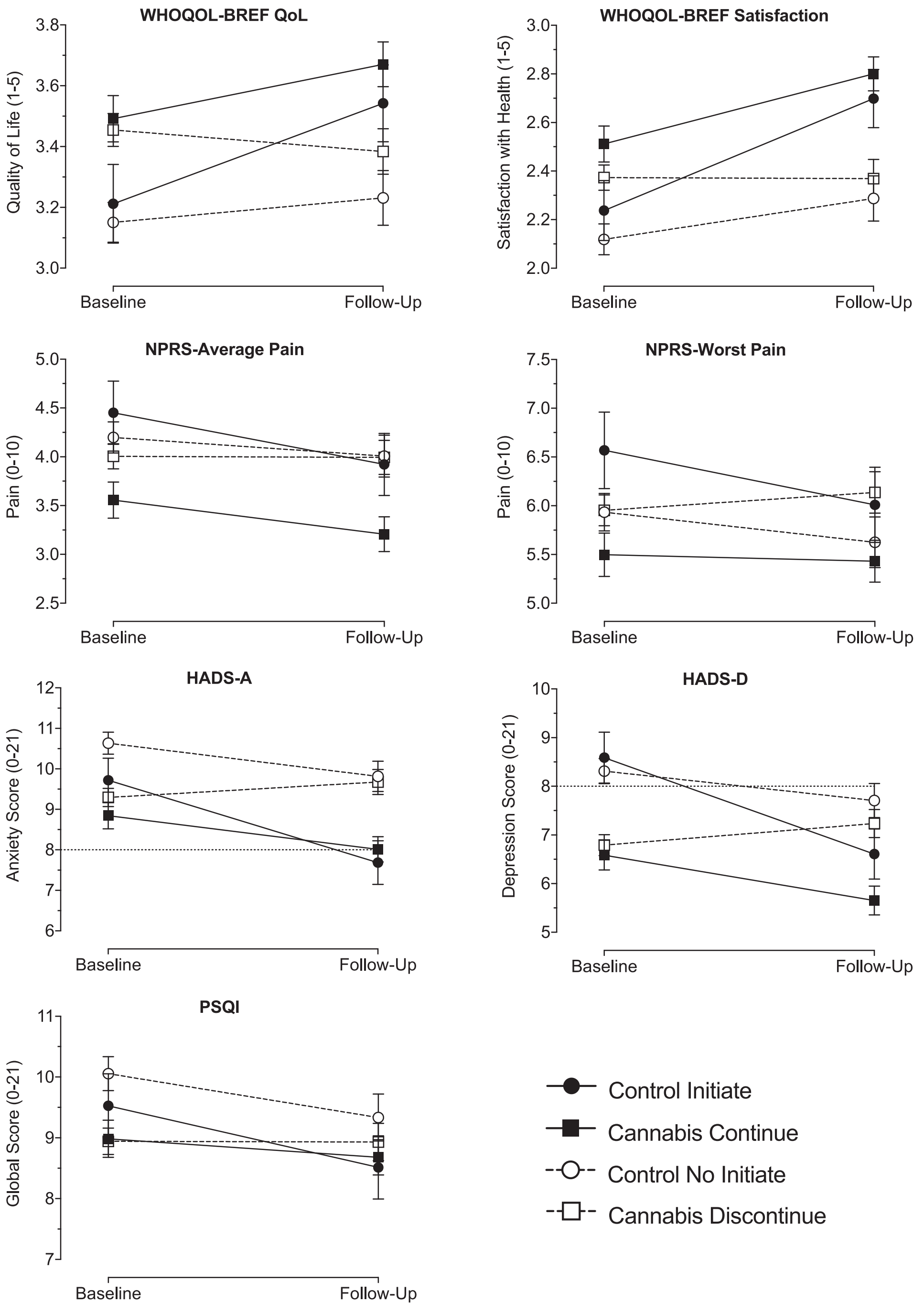

SUPPLEMENTARY FIG. S3. Marginal means for health outcomes at baseline and follow-up by medicinal cannabis use behavior. Plotted are marginal means from the mixed effects model parameterizing cannabis use as time-varying current cannabis use at follow-up only. Follow-up data are presented at the average time since baseline (284 days). Error bars are standard error.

baseline use and report of cannabis use in follow-up (with missing follow-up values treated as nonuse) as well as the interaction of these terms. These Level 2 terms were included to account for potential baseline (intercept) differences in individuals reporting cannabis use at baseline and difference for those who would continue (or initiate) use in follow-up versus those who did not. A final model term evaluated the impact of baseline cannabis use on the time trend effect. We believe that this approach is not preferred over the Person Mean Centering approach described above as it aggregates the possible between- and within-person effects into a single model term ("Current Cannabis Use") making it hard to distinguish these different mechanisms contributing to health indicators. The results of this analysis, nevertheless, were generally consistent with the Person Mean approach insofar as significant effects were observed for all measures (p values < 0.036) with the exception of sleep scores ($p = 0.069$). This pattern likely reflects the contributions of between-person variance in the model term, although we again should note that this pattern still reveals a consistent conclusion for a significant effect of medicinal cannabis for improving health outcomes. Marginal means from the estimated model are presented in Supplementary Figure S2. Sensitivity analyses in which the use in follow-up variable was recoded as last use behavior reported (e.g., cannabis use for the Cannabis Use group and no use for the Control group) revealed a similar significance and magnitude of effect for the impact of current cannabis use on health outcomes as did those using only individuals with follow-up assessments.

Follow-up cannabis use only approach

$$Health\ Outcome_{ij} = \pi_{0i} + FU\ Cannabis\ Use * \pi_{1i} + Time(Days) * \pi_{2i}$$

$$\pi_{0i} = \gamma_{00} + BaselineUse * \gamma_{01} + Use\ in\ FU * \gamma_{02} + Use\ in\ FU * BaselineUse * \gamma_{03} +$$

$$\pi_{1i} = \gamma_{10}$$

$$\pi_{2i} = \gamma_{20} + BaselineUse * \gamma_{21}$$

A final alternative parameterization only treated cannabis use reported in follow-up as a time-varying effect. Other aspects of this model remained the same by incorporating the potential baseline differences and differences in time effects based on baseline use. The results of this parameterization indicated significant effects for quality of life ($p < 0.001$), health satisfaction ($p < 0.001$), average pain ($p = 0.007$), anxiety ($p < 0.001$), and depression ($p < 0.001$). Again, these results are in line with the person mean approach above, and deviations likely reflect aggregation of the between- and within-person effects into a single parameter term for these models. Marginal means from this model parameterization are presented in Supplementary Figure S3.

Supplementary Reference

S1. Wang LP, Maxwell SE. On disaggregating between-person and within-person effects with longitudinal data using multilevel models. Psychol Methods. 2015;20:63–83.
